# Supplementary material for: Deficiency of ASGR1 promotes liver injury by increasing GP73-mediated hepatic endoplasmic reticulum stress
Source: Nat Commun. 2024 Mar 8;15:1908. doi: 10.1038/s41467-024-46135-9 (PMC10924105; doi:10.1038/s41467-024-46135-9)
Supplement: Supplementary file 1 — Supplementary Information [file 41467_2024_46135_MOESM1_ESM.pdf]

Supplementary information

Deficiency of ASGR1 promotes liver injury by increasing GP73-mediated hepatic endoplasmic reticulum stress

Zhe Zhang[1,#], Xiang Kai Leng[1,#], Yuan Yuan Zhai[1,#], Xiao Zhang[1], Zhi Wei Sun[2], Jun Ying Xiao[1], Jun Feng Lu[1], Kun Liu[3], Bo Xia[1], Qi Gao[2], Miao Jia[2], Cheng Qi Xu[4], Yi Na Jiang[5], Xiao Gang Zhang[6,*], Kai Shan Tao[3,*], Jiang Wei Wu[1,*]

[1] Key Laboratory of Animal Genetics, Breeding and Reproduction of Shaanxi Province, College of Animal Science and Technology, Northwest A&F University, Yangling, China.

[2] Beijing Sungen Biomedical Technology Co. Ltd., Beijing, China.

[3] Department of Hepatobiliary Surgery, Xi-Jing Hospital, Air Force Medical University, Xi'an, China.

[4] College of Life Science and Technology, Huazhong University of Science and Technology, Wuhan, China.

[5] Department of Pathology, the First Affiliated Hospital of Xi'an Jiaotong University, Xi'an, China.

[6] Department of Hepatobiliary Surgery, the First Affiliated Hospital of Xi'an Jiaotong University, Xi'an, China.

[#] These authors contributed equally to this work.

[*]Corresponding authors. Email: wujiangwei@nwafu.edu.cn (J.W.);

taokaishan0686@163.com (K.T.);

little_gang17@xjtu.edu.cn (X. Z.);

This file includes:

Supplementary Figures 1 to 20

Supplementary Tables 1 to 3

Fig. S1

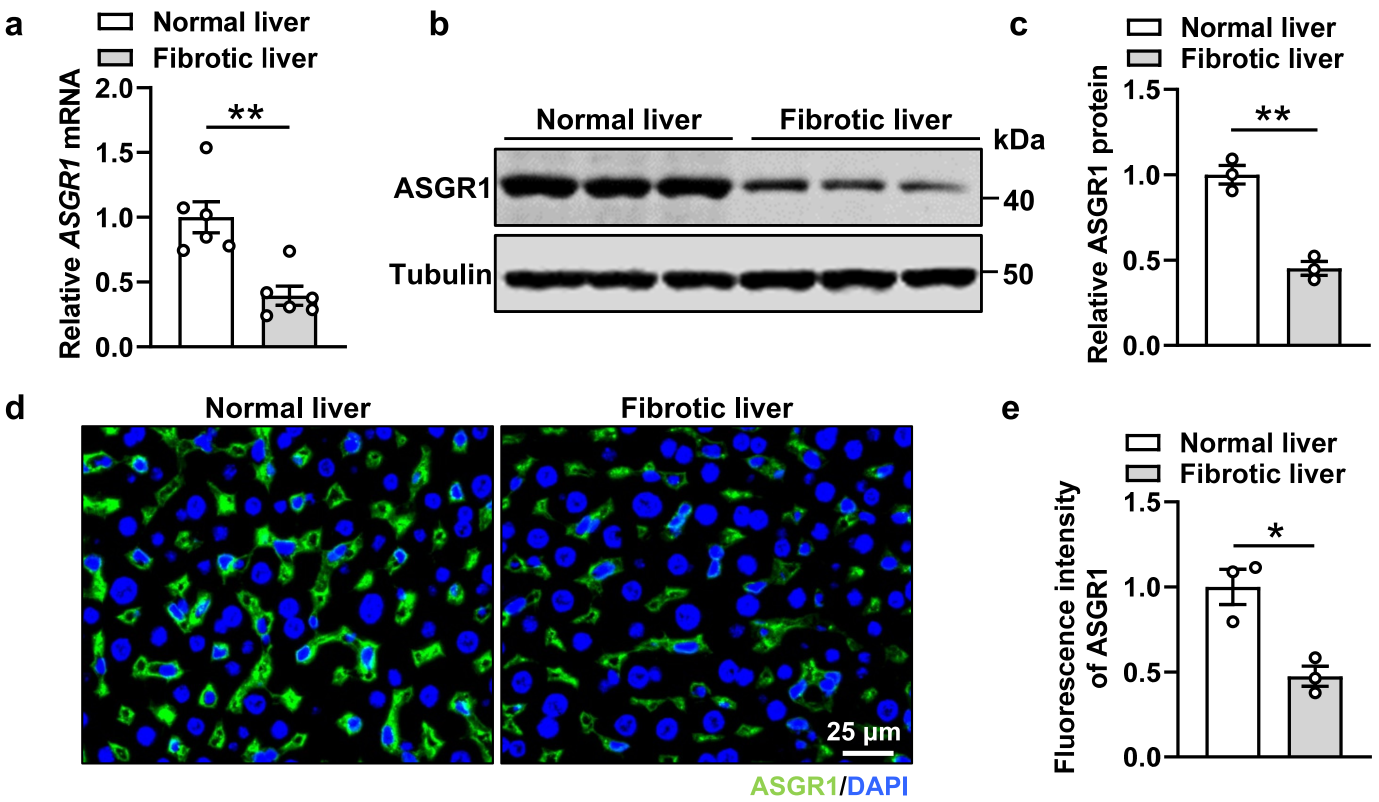

Fig. S1. Hepatic ASGR1 expression is downregulated in patients with liver fibrosis. Liver biopsy specimens were collected from 6 patients with liver fibrosis and 6 normal controls. **(a and b)** Relative mRNA and protein expression of ASGR1. **(c)** Quantification of ASGR1 protein levels (n=3). **(d)** Representative immunofluorescence staining of hepatic ASGR1 in patients with liver fibrosis and normal controls (n=3). Scale bar, 25 μm. **(e)** Quantification of immunofluorescence staining of ASGR1 in patients with liver fibrosis and normal controls. Data are presented as mean ± SEM. *P* values were calculated by two-tailed unpaired t-test. *$P < 0.05$, **$P < 0.01$. Source data are provided as a Source Data file.

Fig. S2

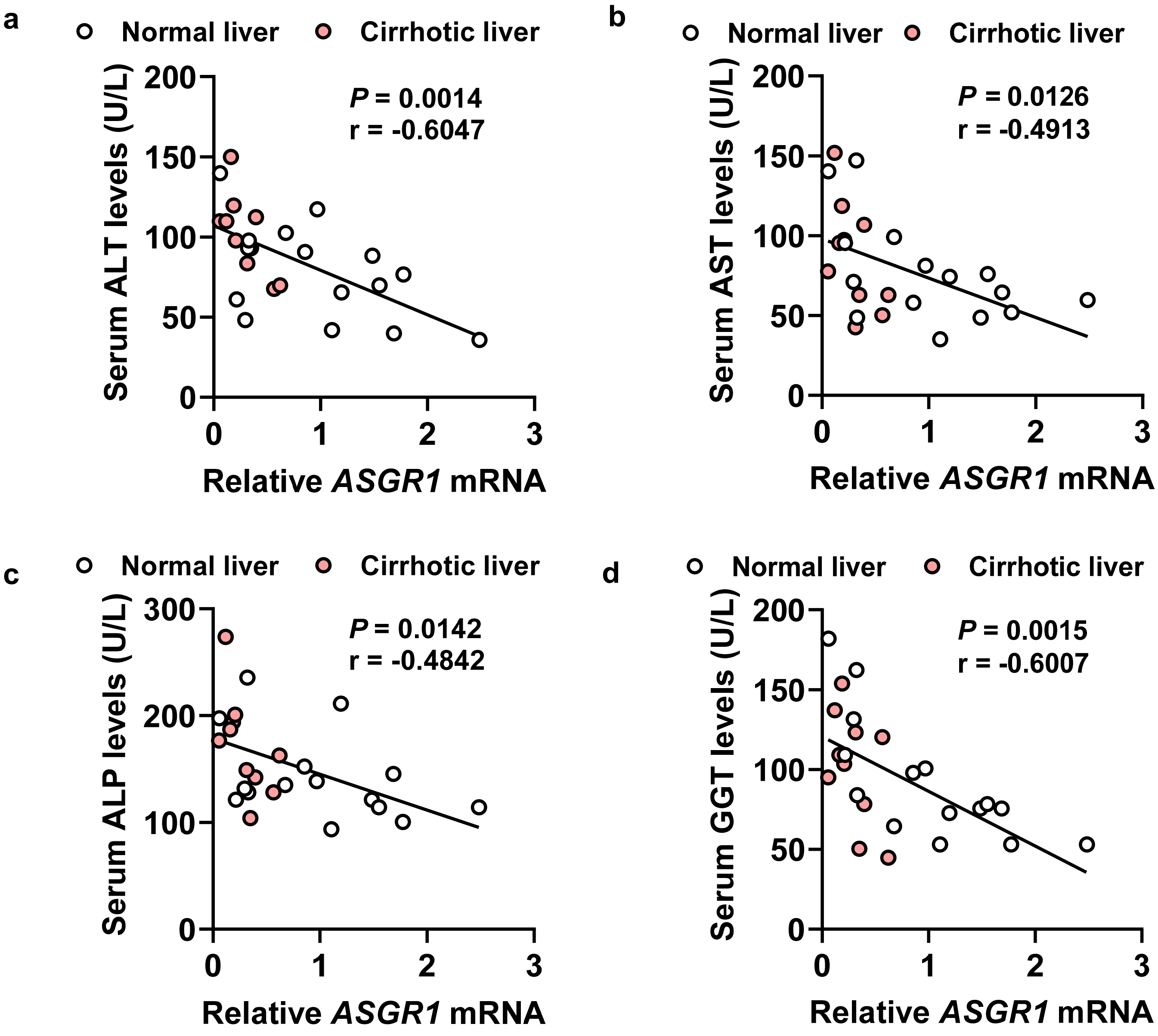

Fig. S2. Correlations between hepatic ASGR1 mRNA and serum levels of liver enzymes in human subjects. Correlations between hepatic ASGR1 mRNA expression and serum ALT **(a)**, AST **(b)**, ALP **(c)** and GGT **(d)** levels in cirrhotic patients (n=10) and normal controls (n=15). Source data are provided as a Source Data file.

Fig. S3

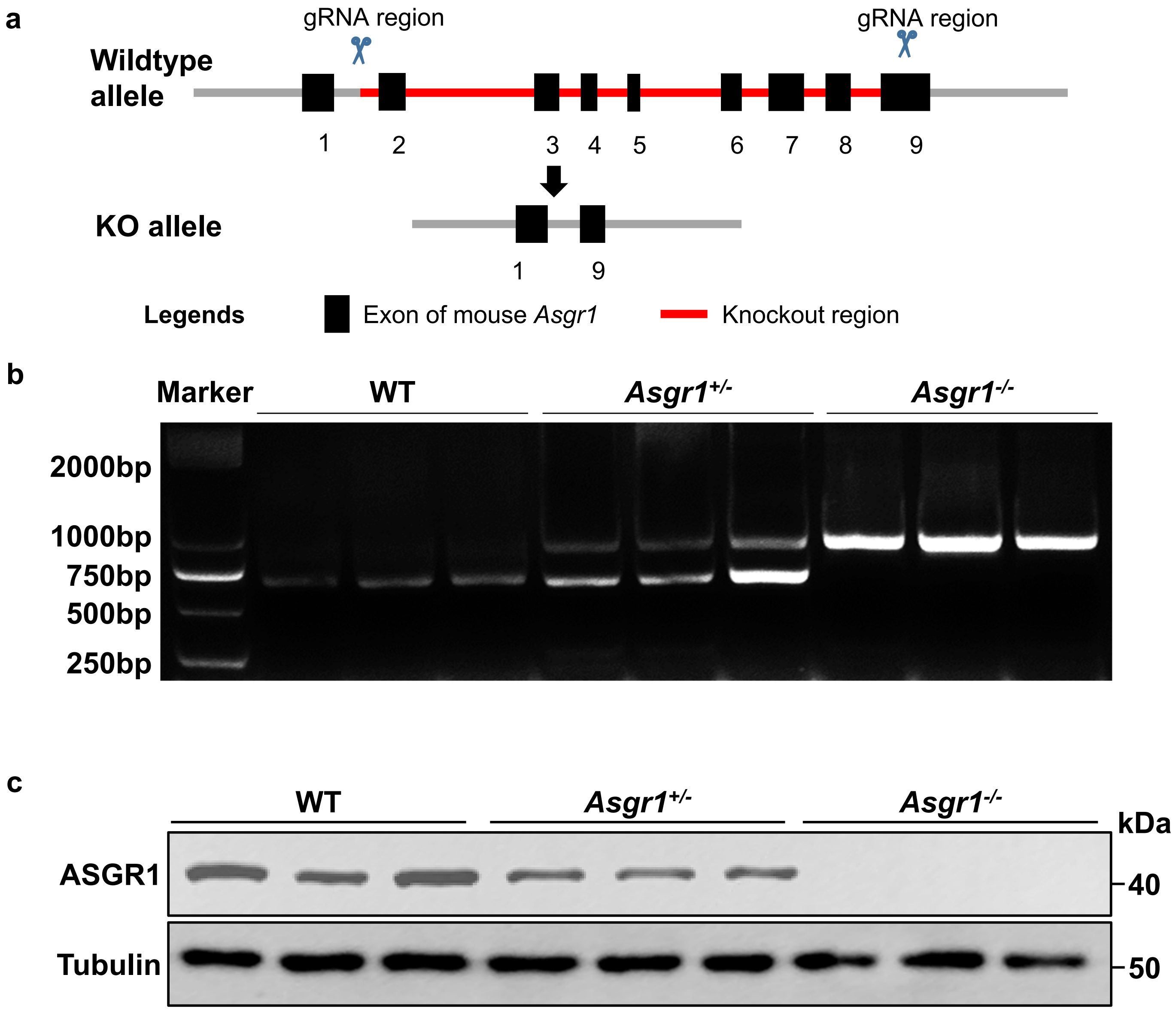

Fig. S3. Generation and characterization of ASGR1-deficient mice. (a) Schematic diagram for the generation of ASGR1-deficient mice using CRISPR/Cas9. **(b)** DNA genotyping of *Asgr1*$^{+/+}$, *Asgr1*$^{+/-}$ and *Asgr1*$^{-/-}$ mice. **(c)** Immunoblotting analysis of ASGR1 in livers of *Asgr1*$^{+/+}$, *Asgr1*$^{+/-}$ and *Asgr1*$^{-/-}$ mice. Source data are provided as a Source Data file.

Fig. S4

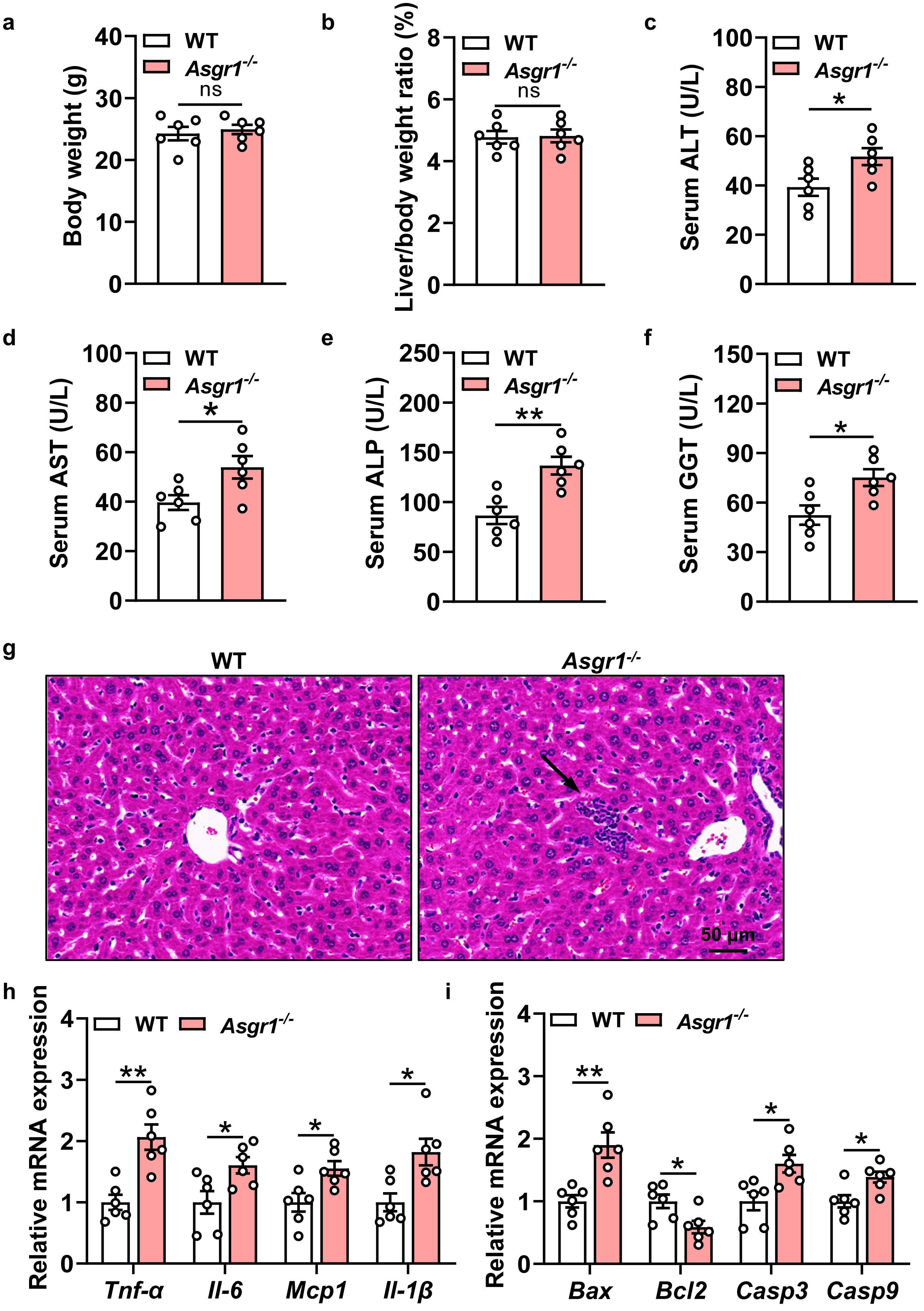
a
Body weight (g)
WT
Asgr1-/-
ns
b
Liver/body weight ratio (%)
c
Serum ALT (U/L)
*
d
Serum AST (U/L)
e
Serum ALP (U/L)
**
f
Serum GGT (U/L)
g
WT
Asgr1-/-
50 μm
h
Relative mRNA expression
Tnf-α
Il-6
Mcp1
Il-1β
i
Bax
Bcl2
Casp3
Casp9

Fig. S4. ASGR1-deficient mice show liver injury under normal conditions. Six-month-old *Asgr1*$^{-/-}$ mice and their WT controls were randomly grouped under normal diet (n = 6). **(a)** Body weight. (b) Liver-to-body weight ratio. **(c to f)** Serum levels of ALT, AST, ALP and GGT. **(g)** H&E staining of liver sections. Scale bars, 50 μm. **(h)** Relative hepatic mRNA expression of the proinflammatory genes. **(i)** Relative hepatic mRNA expression of the apoptosis-related genes. Data are presented as mean ± SEM. *P* values were calculated by two-tailed unpaired t-test. **P* < 0.05, ***P* < 0.01. Source data are provided as a Source Data file.

Fig. S5

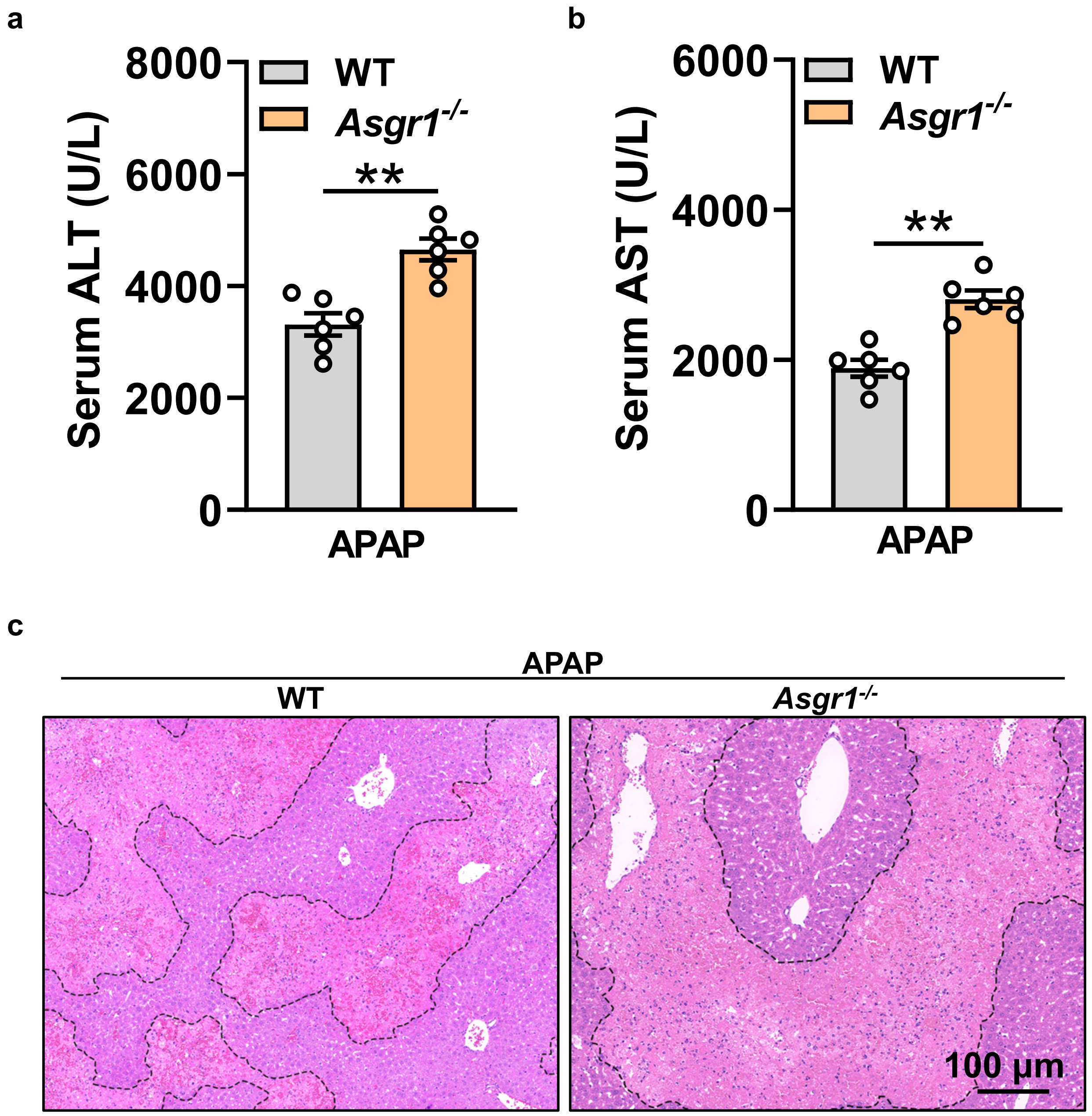

Fig. S5. ASGR1 deficiency aggravates acute liver injury. 8-week-old WT and *Asgr1*$^{-/-}$ mice were intraperitoneally injected with APAP (400mg/kg body weight) (n = 6). **(a and b)** Serum levels of ALT and AST in mice after 24 hours. **(c)** H&E staining of liver sections. Scale bars, 100 µm. Necrotic areas were encircled. Data are presented as mean ± SEM. *P* values were calculated by two-tailed unpaired t-test. *$P < 0.05$, **$P < 0.01$. Source data are provided as a Source Data file.

Fig. S6

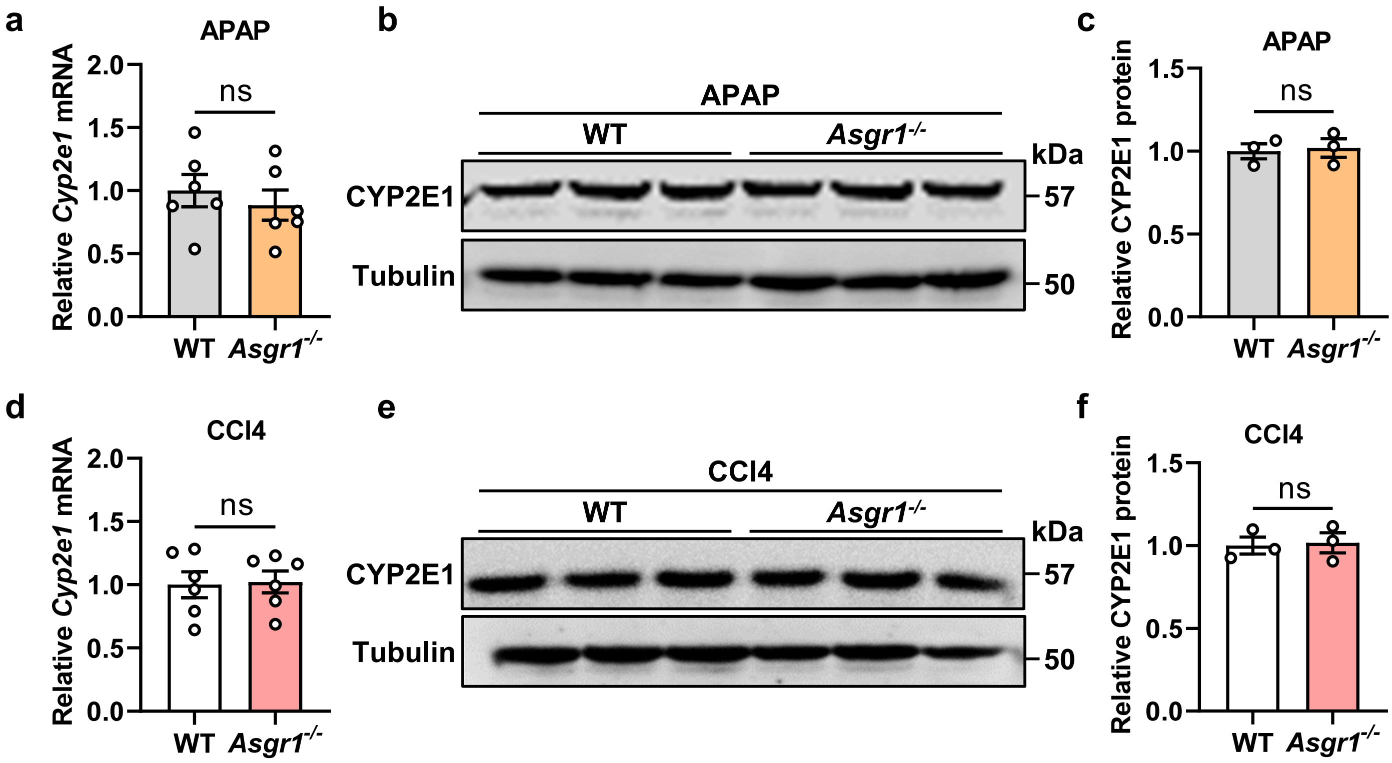

Fig. S6. ASGR1 deficiency does not affect CYP2E1 expression in liver injured mice. (a and b) Relative hepatic mRNA and protein expression of CYP2E1 in WT or *Asgr1*$^{-/-}$ mice treated with APAP. **(c)** Quantification of CYP2E1 protein levels (n=3). **(d and e)** Relative hepatic mRNA and protein expression of CYP2E1 in WT or *Asgr1*$^{-/-}$ mice treated with CCl4. **(f)** Quantification of CYP2E1 protein levels (n=3). Data are presented as mean ± SEM. *P* values were calculated by two-tailed unpaired t-test. **P* < 0.05, ***P* < 0.01. Source data are provided as a Source Data file.

Fig. S7

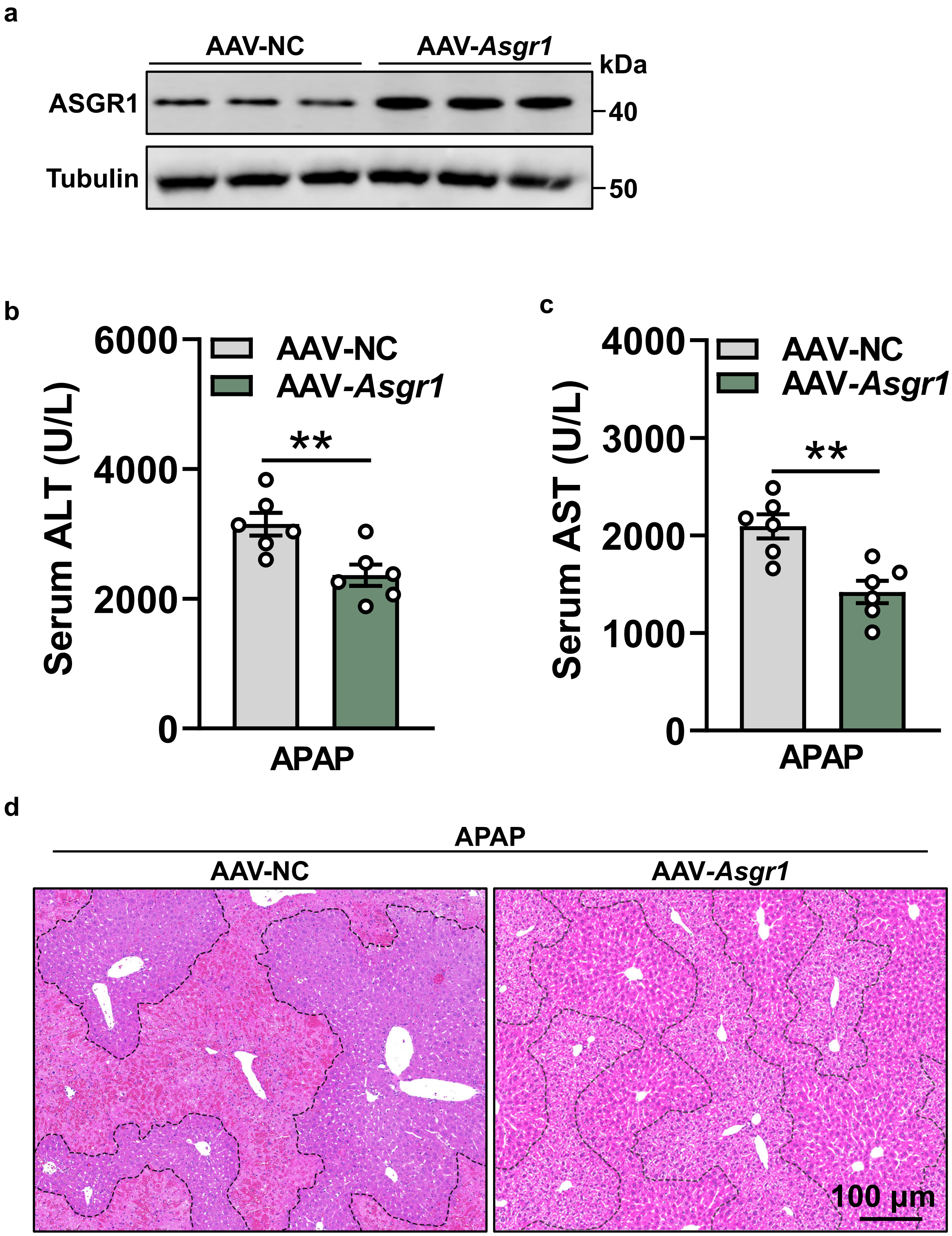

Fig. S7. Hepatic overexpression of *ASGR1* protects against APAP-induced acute liver injury. 8-week-old mice were treated with AAV-*Asgr1* or AAV-NC for 4 weeks and then intraperitoneally injected with APAP (400mg/kg body weight) for 24 hours (n=6). **(a)** Representative immunoblotting analysis of hepatic ASGR1. **(b and c)** Serum levels of ALT and AST. **(d)** H&E staining of liver sections. Scale bars, 100 µm. Necrotic areas were encircled. Data are presented as mean ± SEM. *P* values were calculated by two-tailed unpaired t-test. **P* < 0.05, ***P* < 0.01. Source data are provided as a Source Data file.

Fig. S8

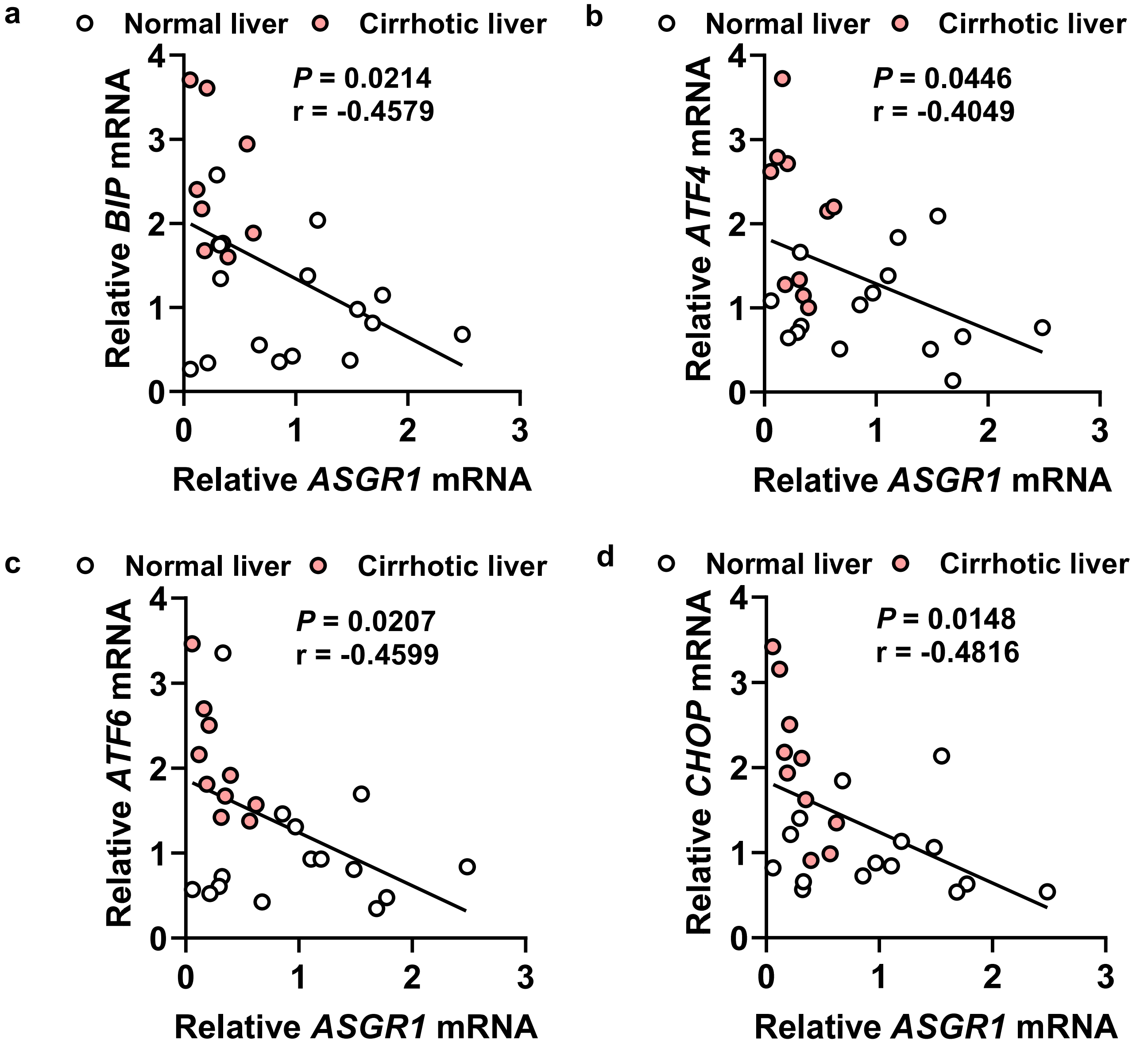

Fig. S8. Correlations between mRNA expression of hepatic *ASGR1* and ER stress markers in human subjects. Correlations between hepatic *ASGR1* mRNA and hepatic BIP **(a)**, ATF4 **(b)**, ATF6 **(c)** and CHOP **(d)** mRNA expression in cirrhotic patients (n=10) and normal controls (n=15). Source data are provided as a Source Data file.

Fig. S9

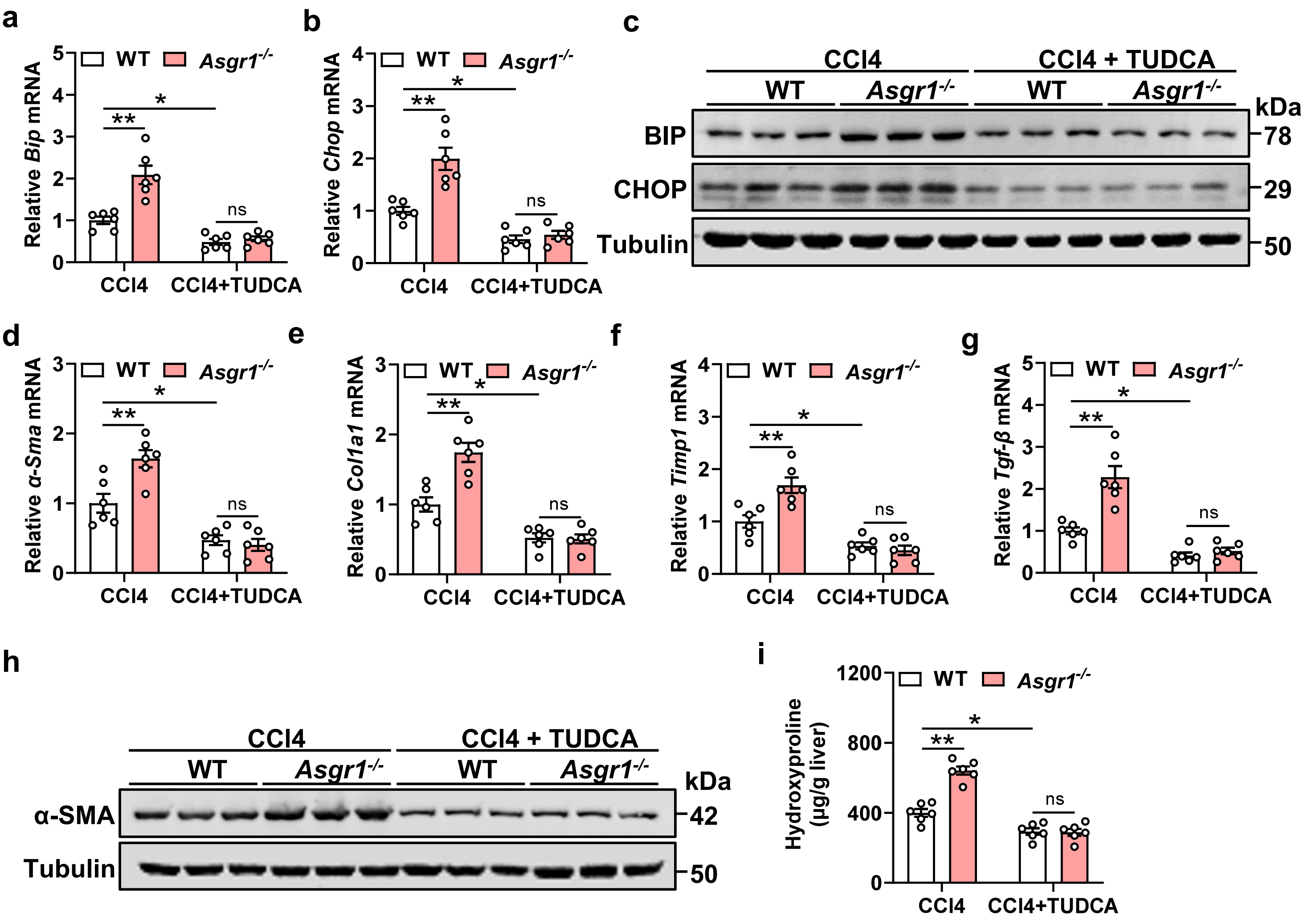

Fig. S9. Pharmacological inhibition of ER stress attenuates ASGR1 deficiency-induced liver injury in CCl4-treated mice. 8-week-old *Asgr1*$^{-/-}$ and WT mice were intraperitoneally injected with CCl4 (1ml/kg body weight, twice a week for 6 weeks). During the last 4 weeks, mice were received either an ER stress inhibitor TUDCA (500 mg/kg body weight, every two days) or vehicle (n=6). **(a to c)** Relative hepatic mRNA and protein expression of BIP and CHOP. **(d to g)** Relative hepatic mRNA expression of the profibrotic genes (n=6). **(h)** Representative immunoblotting analysis of hepatic α-SMA. **(i)** Hepatic hydroxyproline content. Data are presented as mean ± SEM. *P* values were calculated by two-way ANOVA with Tukey's multiple comparison test. **P* < 0.05, ***P* < 0.01. Source data are provided as a Source Data file.

Fig. S10

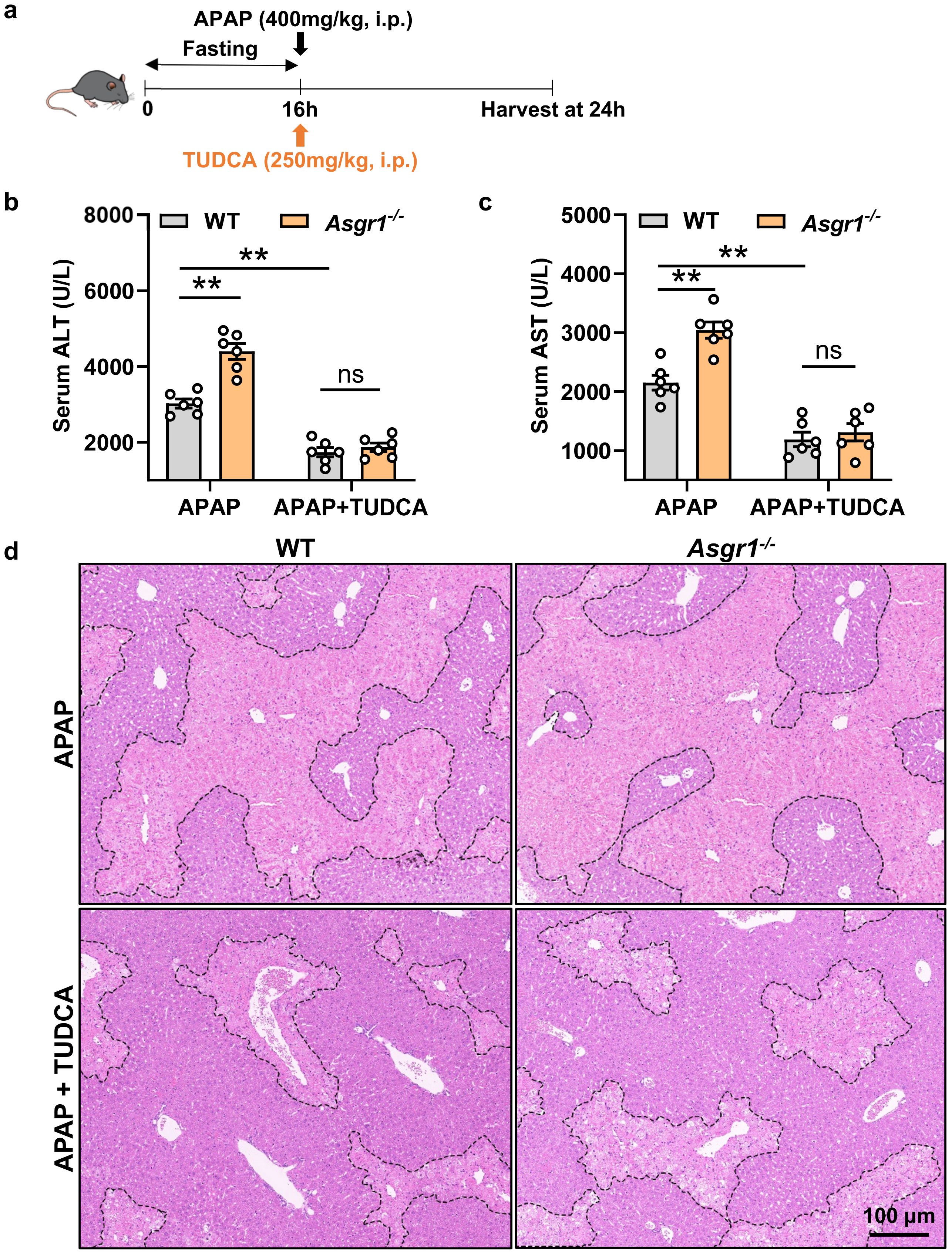

Fig. S10. Pharmacological inhibition of ER stress attenuates ASGR1 deficiency-induced liver injury in APAP-treated mice. (a) Schematic diagram of mice treatment. 8-week-old *Asgr1*$^{-/-}$ and WT mice with APAP intoxication (400mg/kg body weight) were treated with TUDCA (250 mg/kg body weight) or vehicle (n=6). **(b and c)** Serum levels of ALT and AST. **(d)** H&E staining of liver sections. Scale bars, 100 μm. Necrotic areas were encircled. Data are presented as mean ± SEM. *P* values were calculated by two-way ANOVA with Tukey's multiple comparison test. *P < 0.05, **P < 0.01**.** Source data are provided as a Source Data file.

Fig. S11

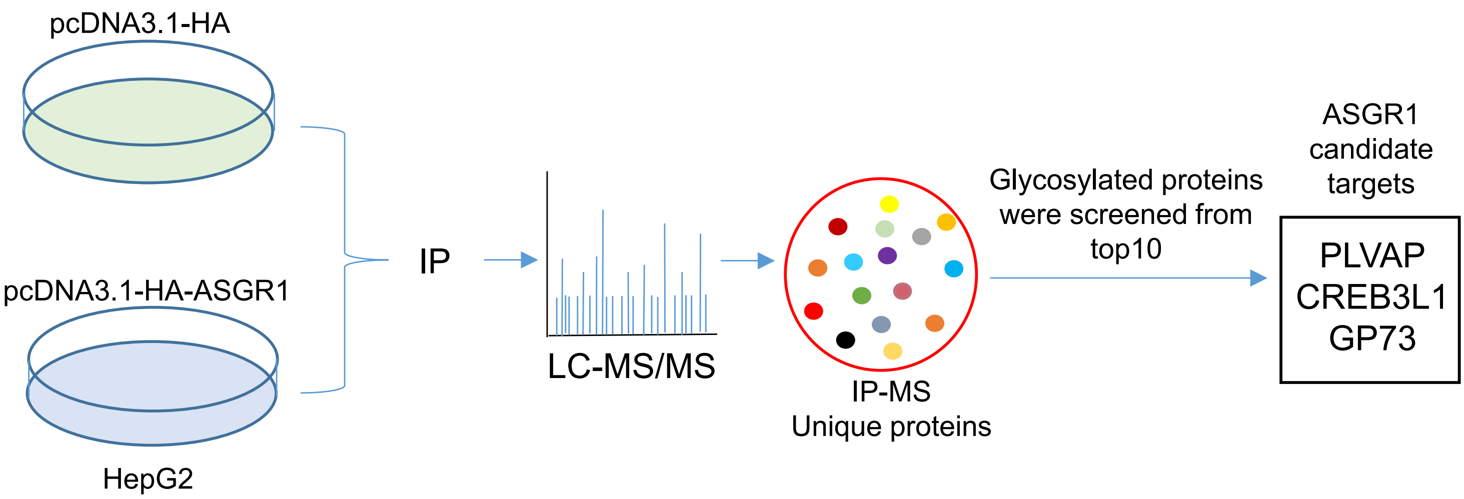

Fig. S11. The flowchart of IP-MS analysis. The HA-tagged ASGR1 protein complexes were isolated in HepG2 cells by immunoprecipitation and subsequently the immunoprecipitates were analyzed by LC-MS/MS. Further screening of glycoprotein ligands with structural characteristics of ASGR1 ligands (containing terminal non-reducing galactose residues and Nacetylgalactosamine residues of tri- or tetra-antennary N-linked glycans) was performed using an online tool (https://www.uniprot.org/). Three proteins PLVAP, CREB3L1 and GP73 were identified as potential ligands for ASGR1.

Fig. S12

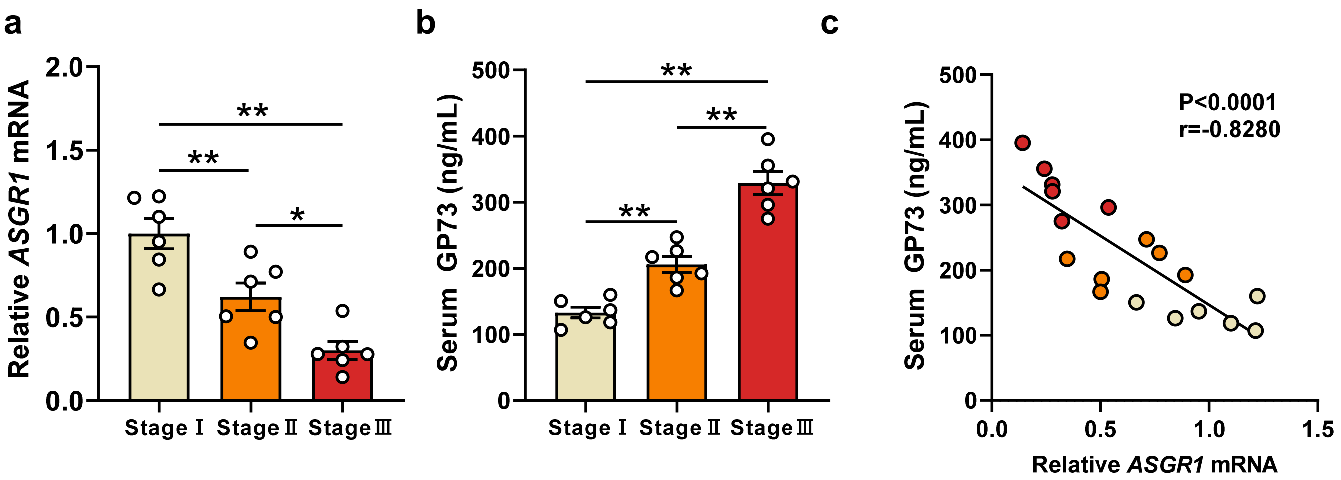

Fig. S12. Hepatic *ASGR1* mRNA expression was negatively correlated with serum levels of GP73 in patients with hepatocellular carcinoma. (a) Relative hepatic mRNA expression of *ASGR1* (n=6). **(b)** Serum levels of GP73 (n=6). **(c)** Correlation between hepatic *ASGR1* mRNA expression and serum GP73 levels in patients with hepatocellular carcinoma. Data are presented as mean ± SEM. *P* values were calculated by one-way ANOVA with Tukey's multiple comparisons test. *$P < 0.05$, **$P < 0.01$. Source data are provided as a Source Data file.

Fig. S13

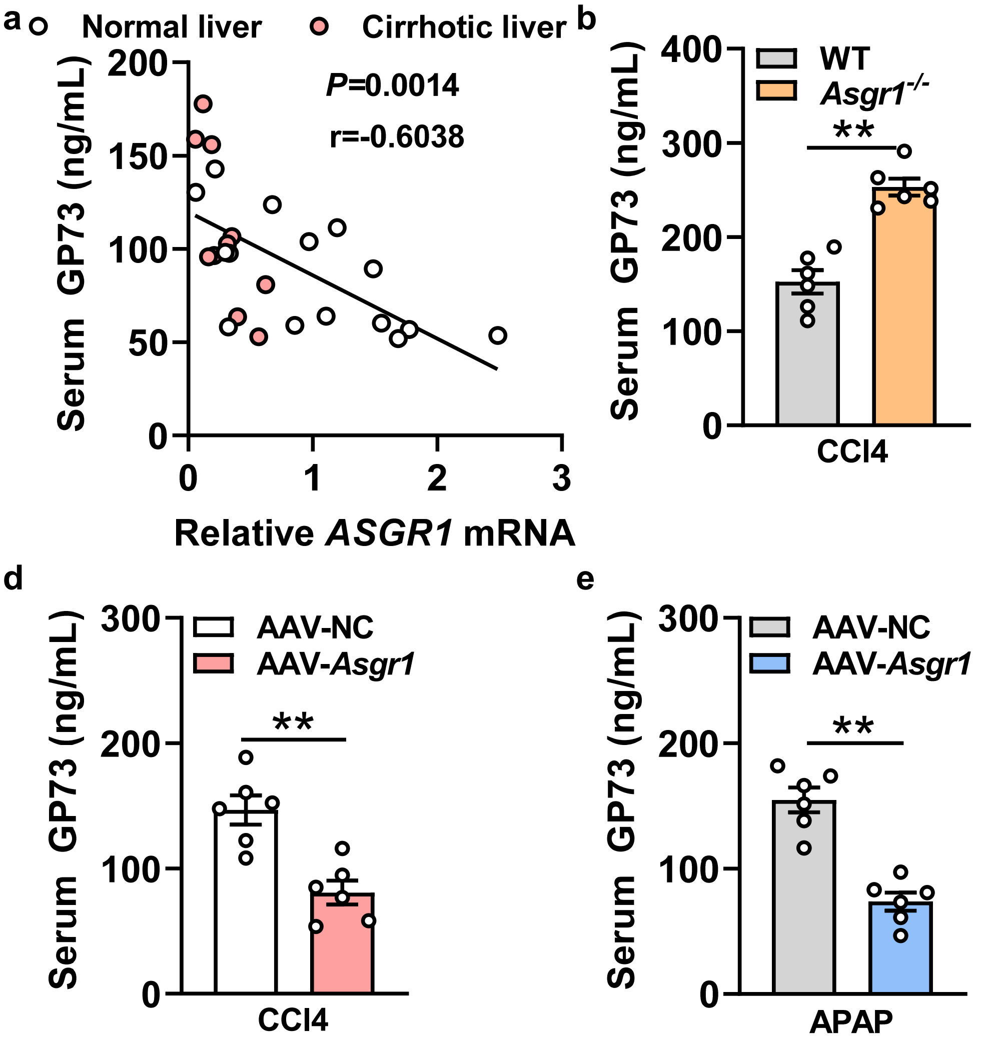

Fig. S13. ASGR1 regulates serum GP73 levels. (a) Correlation between hepatic *ASGR1* mRNA expression and serum GP73 levels in cirrhotic patients (n=10) and normal controls (n=15). **(b and c)** Serum levels of GP73 in WT and *Asgr1*$^{-/-}$ mice treated with CCl4 **(b)** or APAP **(c)**. **(d and e)** Serum levels of GP73 in mice treated with AAV-*Asgr1* or AAV-NC upon CCl4 or APAP treatment. Data are presented as mean ± SEM. *P* values were calculated by two-tailed unpaired t-test. **P* < 0.05, ***P* < 0.01. Source data are provided as a Source Data file.

Fig. S14

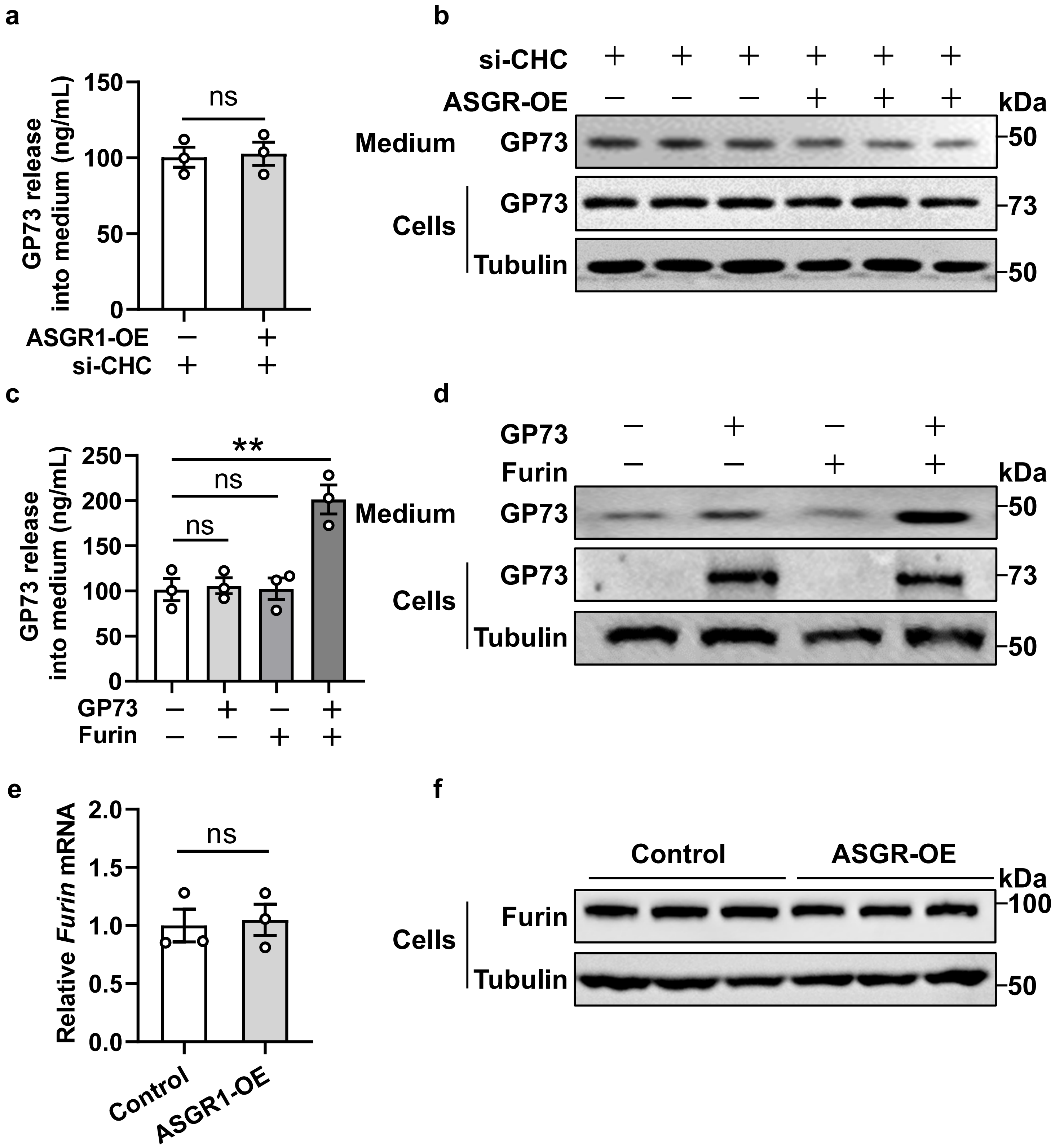

Fig. S14. Hepatic ASGR1 is not involved in GP73 release. (a) Medium GP73 levels in control or *ASGR1*-overexpressing HepG2 cells transfected with si-*CHC* (n=3). **(b)** Relative protein levels of GP73 in cells or medium of control or *ASGR1*-overexpressing HepG2 cells transfected with si-*CHC* (n=3). **(c)** Medium GP73 levels in control or *GP73*-overexpressing HepG2 cells transfected with or without *Furin* (n=3). **(d)** Relative protein levels of GP73 in cells or medium of control or *GP73*-overexpressing HepG2 cells in the presence or absence of *Furin* overexpression. **(e and f)** Relative mRNA and protein expression of Furin in control or *ASGR1*-overexpressing HepG2 cells (n=3). Data are presented as mean ± SEM. *P* values were calculated by two-tailed unpaired t-test (a, e), or one-way ANOVA with Dunnett's multiple comparisons test (c). **P* < 0.05, ***P* < 0.01. Source data are provided as a Source Data file.

Fig. S15

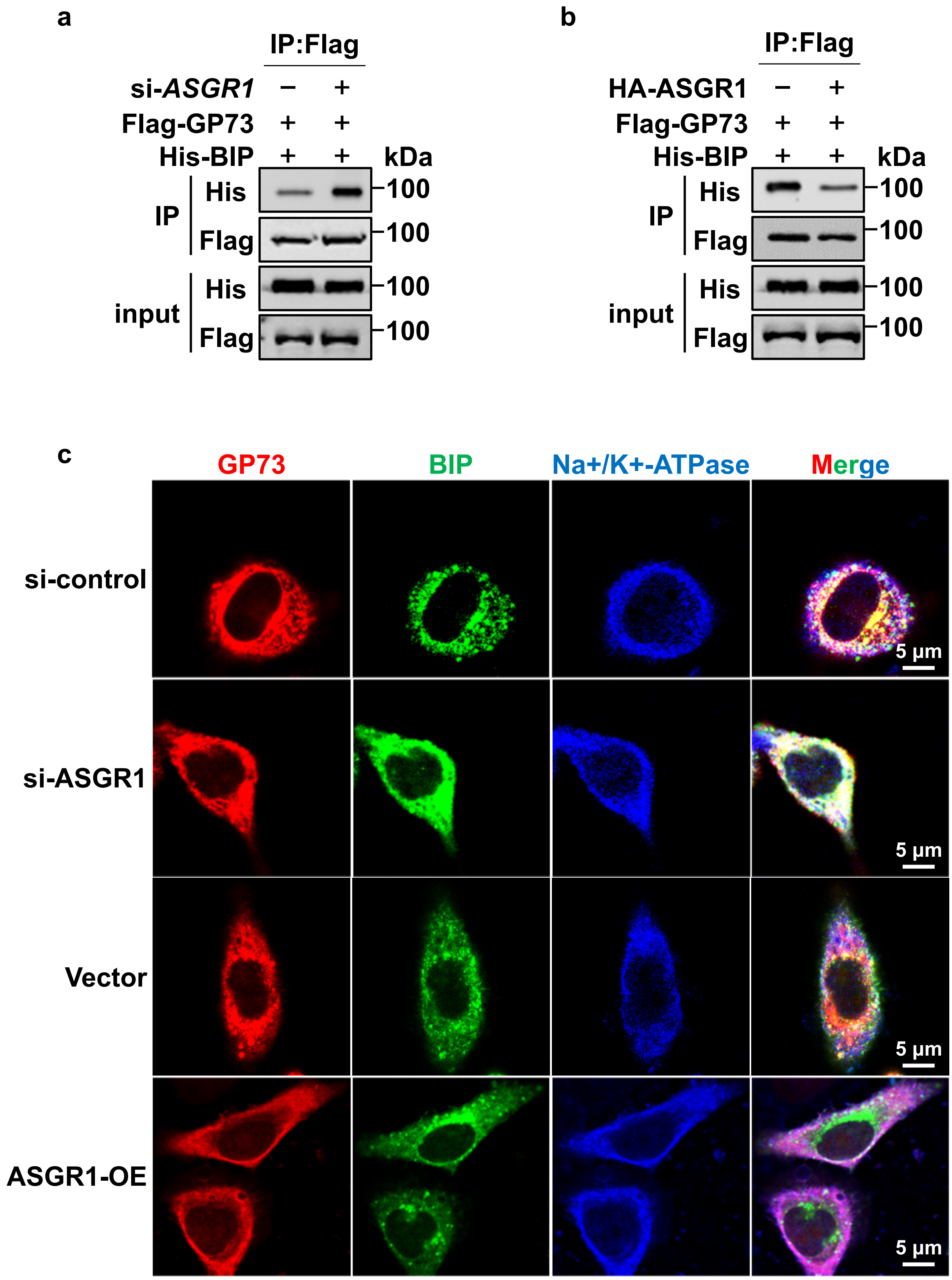

Fig. S15. ASGR1 regulates the binding of BIP to GP73. (a) The interaction between GP73 and BIP in HepG2 cells transfected with si-control or si-*ASGR1*. **(b)** The interaction between GP73 and BIP in HepG2 cells transfected with vector or HA-ASGR1. **(c)** Representative confocal immunofluorescence of GP73 colocalization with BIP at the plasma membrane of *ASGR1*-knockdown or *ASGR1*-overexpressing HepG2 cells. Red represents GP73, green represents BIP, and blue represents the plasma membrane. Scale bars, 5 μm. Source data are provided as a Source Data file.

Fig. S16

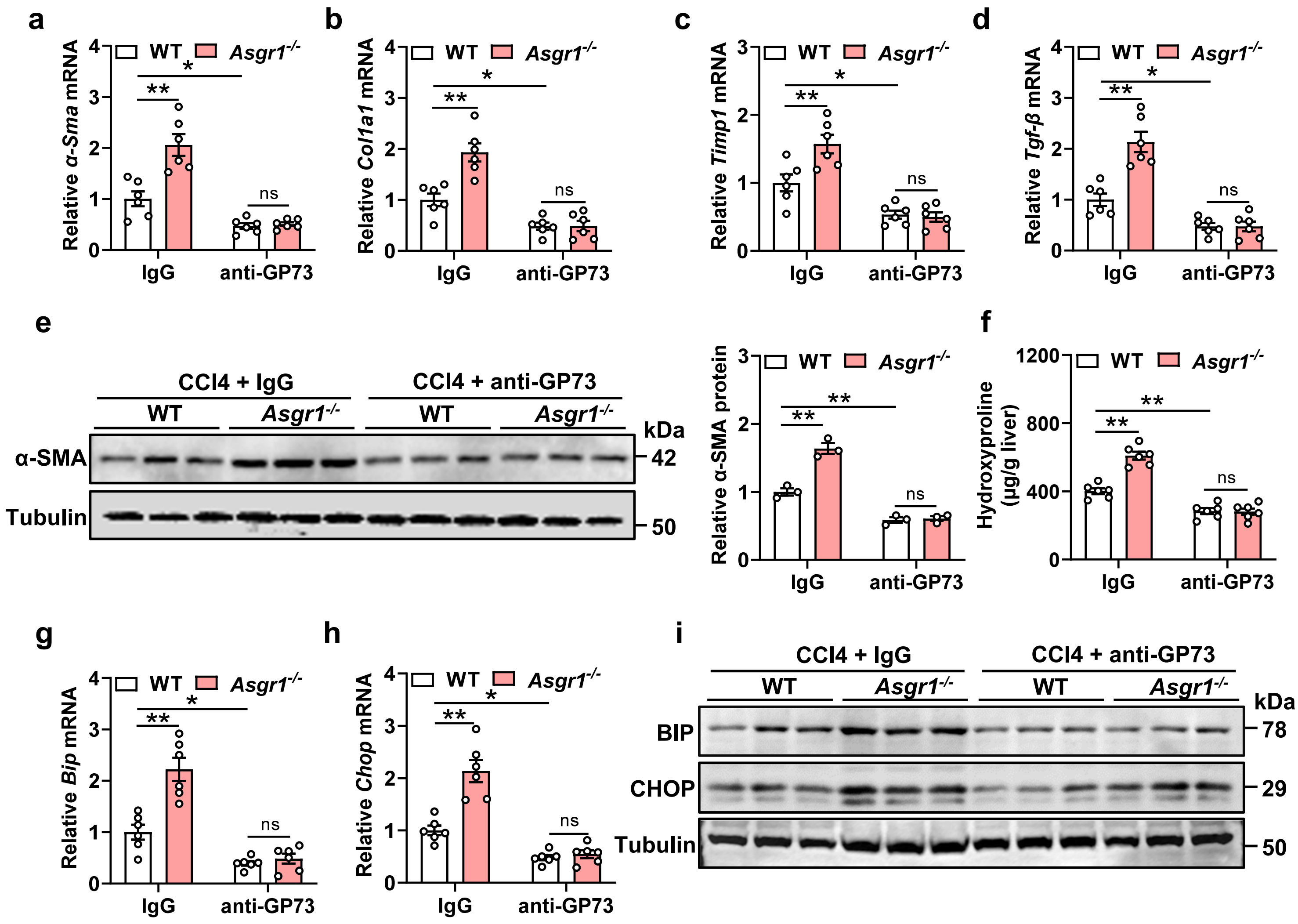

Fig. S16. GP73 neutralization attenuates ASGR1 deficiency-induced liver injury in CCl4-treated mice. 8-week-old *Asgr1*$^{-/-}$ and WT mice were intraperitoneally injected with CCl4 (1ml/kg body weight, twice a week for 6 weeks). During the last 4 weeks, mice were received either anti-GP73 (10mg/kg body weight, twice a week for 4 weeks) or IgG (n=6). **(a to d)** Relative mRNA expression of profibrotic genes in the liver. **(e)** Representative immunoblotting analysis of α-SMA and its quantification in the liver (n=3). **(f)** Hepatic hydroxyproline content. **(g and h)** Relative hepatic mRNA expression of *Bip* and *Chop*. **(i)** Relative hepatic protein expression of BIP and CHOP. Data are presented as mean ± SEM. *P* values were calculated by two-way ANOVA with Tukey's multiple comparison test. *$P < 0.05$, **$P < 0.01$. Source data are provided as a Source Data file.

Fig. S17

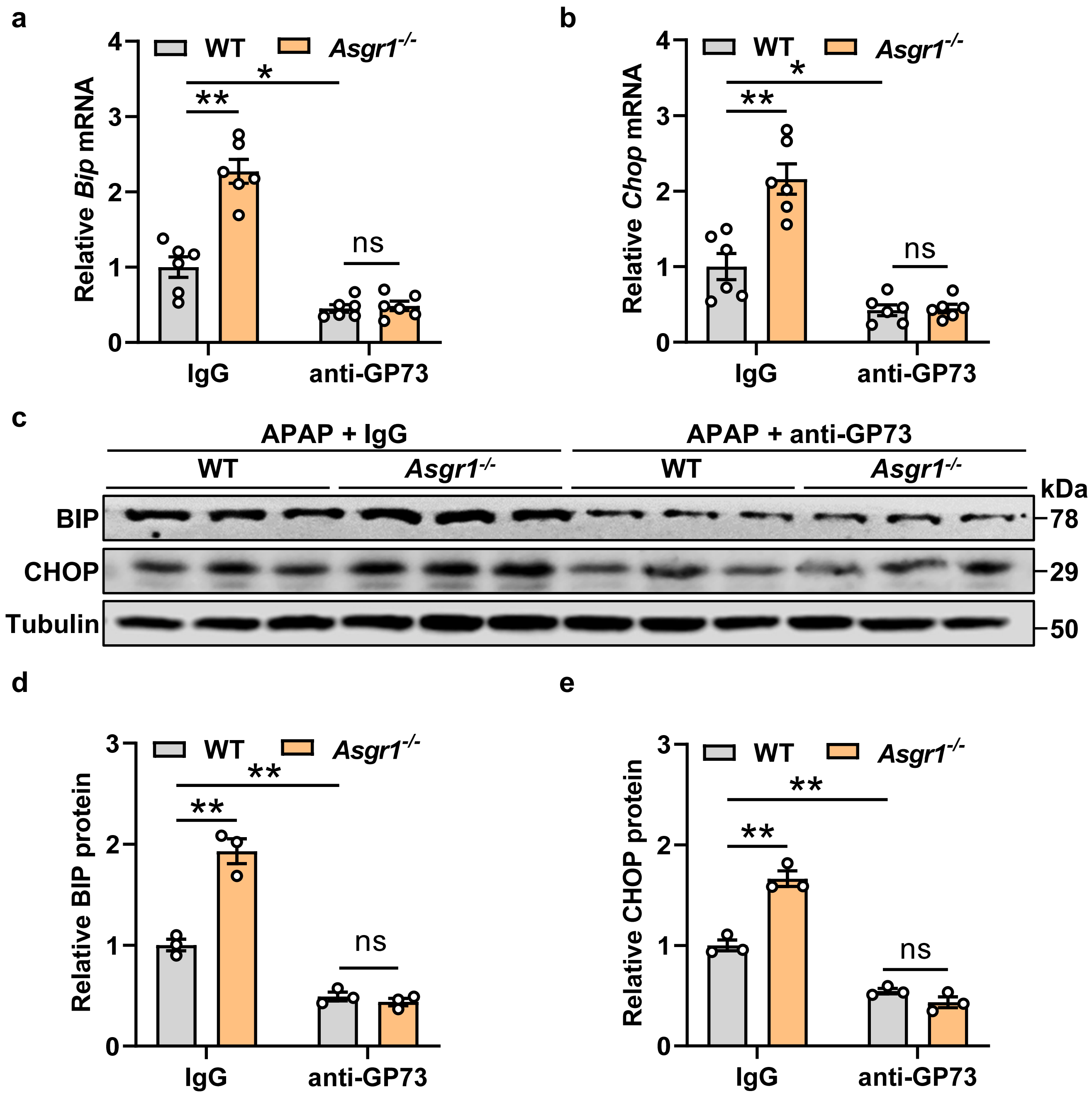

Fig. S17. GP73 neutralization attenuates ASGR1 deficiency-induced ER stress in APAP-treated mice. 8-week-old *Asgr1*$^{-/-}$ and WT mice were intraperitoneally injected with anti-GP73 (10mg/kg body weight) or IgG 10 hours after APAP injection (400mg/kg body weight) (n=6). **(a to c)** Relative hepatic mRNA and protein expression of BIP and CHOP. **(d and e)** Quantification of BIP and CHOP protein levels (n=3). Data are presented as mean ± SEM. *P* values were calculated by two-way ANOVA with Tukey's multiple comparison test. *$P < 0.05$, **$P < 0.01$. Source data are provided as a Source Data file.

Fig. S18

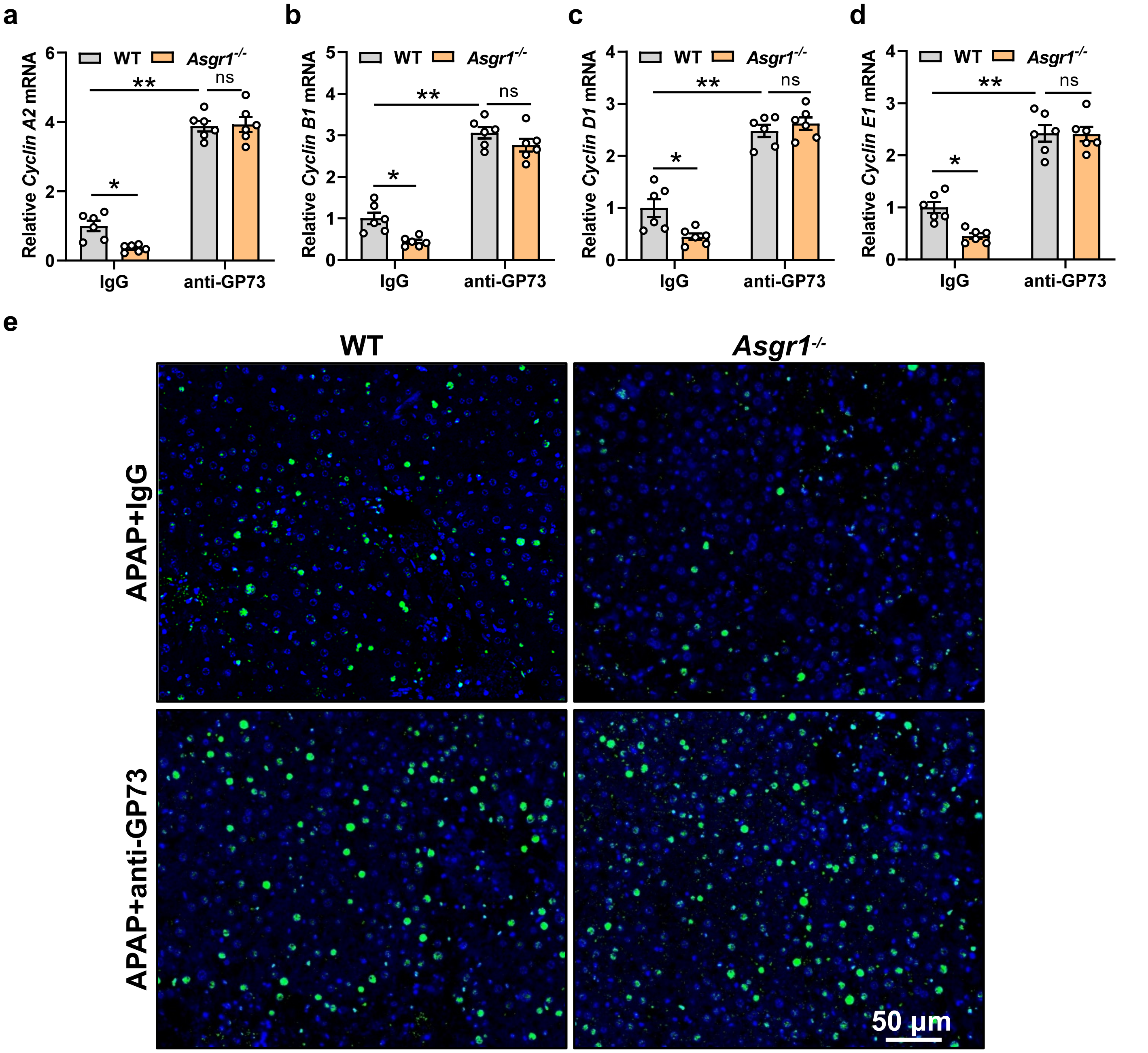

Fig. S18. GP73 neutralization rescues ASGR1 deficiency-induced impaired liver regeneration in APAP-treated mice. 8-week-old *Asgr1*$^{-/-}$ and WT mice were intraperitoneally injected with anti-GP73 (10mg/kg body weight) or IgG 10 hours after APAP injection (400mg/kg body weight) (n=6). **(a to d)** Relative hepatic mRNA expression of *cyclin A2/B1/D1/E1*. **(e)** Representative images of Ki-67 immunofluorescent staining. Scale bars, 50 µm. Data are presented as mean ± SEM. *P* values were calculated by two-way ANOVA with Tukey's multiple comparison test. *$P < 0.05$, **$P < 0.01$. Source data are provided as a Source Data file.

Fig. S19

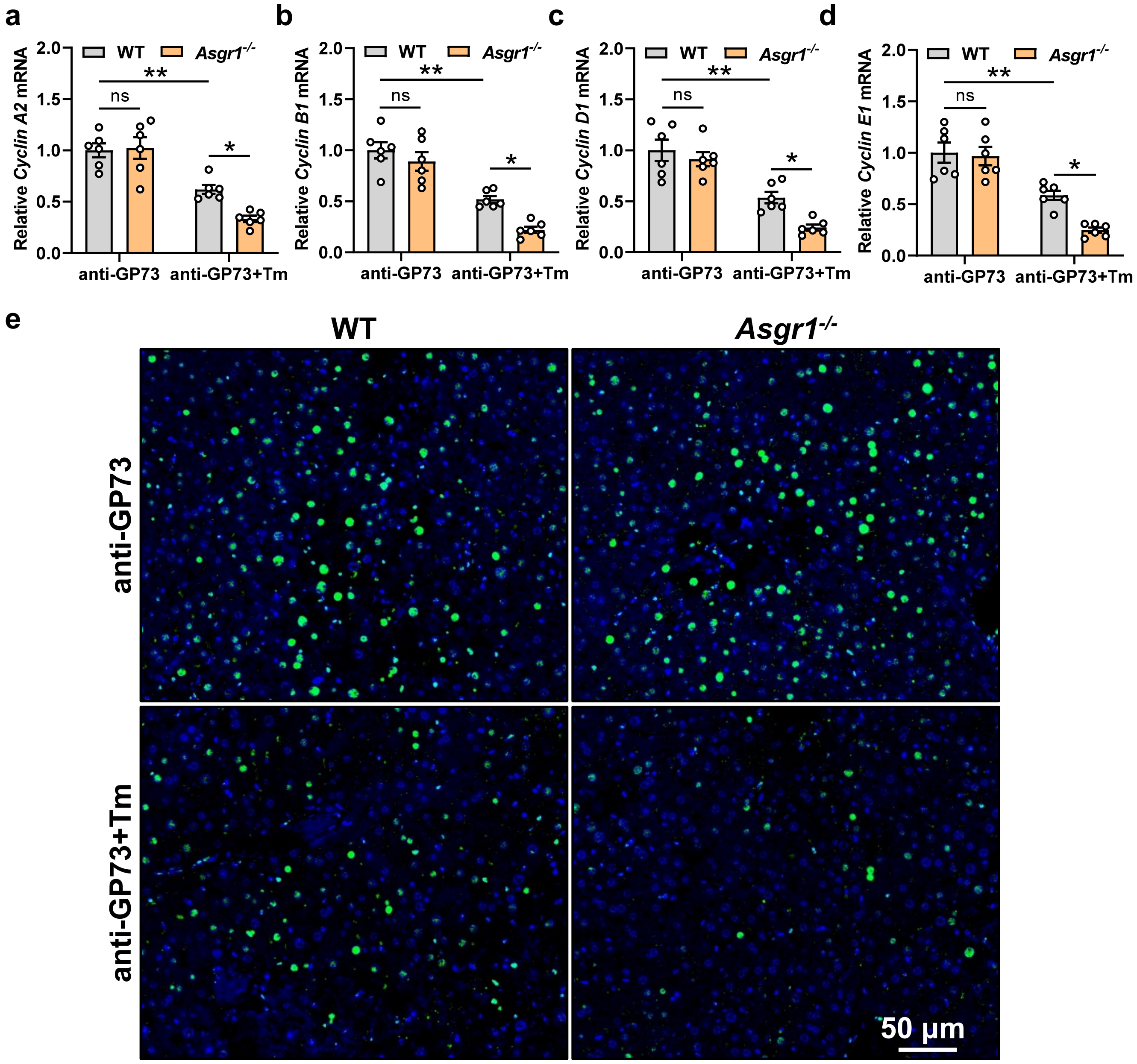

Fig. S19. ER stress agonist inhibits anti-GP73 induced liver regeneration. 8-week-old *Asgr1*$^{-/-}$ and WT mice intraperitoneally injected with anti-GP73 (10mg/kg body weight) were treated with Tm (2mg/kg body weight) or vehicle 10 hours after APAP injection (400mg/kg body weight) (n=6). **(a to d)** Relative hepatic mRNA expression of *cyclin A2/B1/D1/E1*. **(e)** Representative images of Ki-67 immunofluorescent staining. Scale bars, 50 μm. Data are presented as mean ± SEM. *P* values were calculated by two-way ANOVA with Tukey's multiple comparison test. **P* < 0.05, ***P* < 0.01. Source data are provided as a Source Data file.

Fig. S20

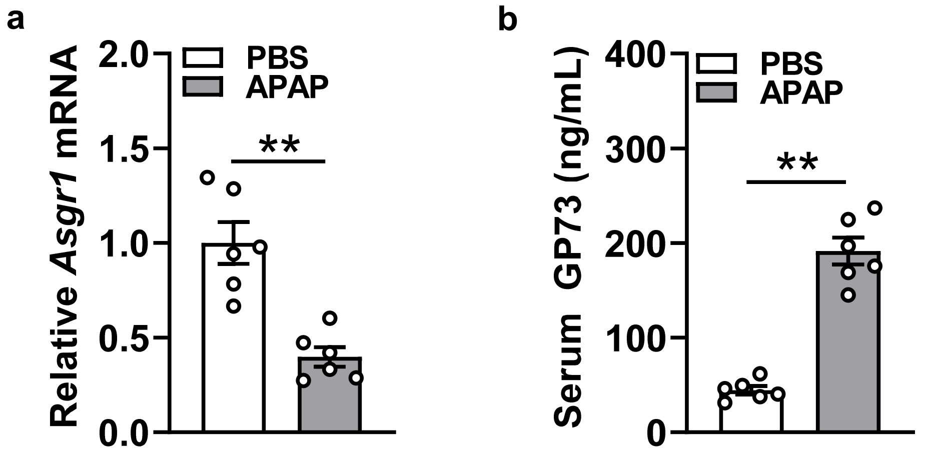

Fig. S20. Hepatic *Asgr1* expression and serum GP73 levels in lethal dose of APAP-treated mice. 8-week-old mice were intraperitoneally injected with a lethal dose of APAP (650 mg/kg body weight) or vehicle (n=6). **(a)** Relative hepatic mRNA expression of *Asgr1*. **(b)** Serum levels of GP73. Data are presented as mean ± SEM. *P* values were calculated by two-tailed unpaired t-test. **P* < 0.05, ***P* < 0.01. Source data are provided as a Source Data file.

Table S1. Clinical characteristics of all patients in this study

Characteristic	n=49
**Sex**	
Female	24
Male	25
**Age (years) (Mean±SD)**	51.78±12.98 (30-78)
**Liver Disease**	
Normal	15
Liver fibrosis	6
Liver cirrhosis	10
hepatocellular carcinoma	18
**pTNM stage**	
I	6
II	6
III	6

Table S2. qRT-PCR primers used in this study

Species	Gene	Primers	Size (bp)	Accession Number
Mouse	*Tnf-α*	CCACCACGCTCTTCTGTCT	180	NM_013693.3
		GCTCCTCCACTTGGTGGTTT		
	Il6	CTCATTCTGCTCTGGAGCCC	89	NM_031168.2
		CAACTGGATGGAAGTCTCTTGC		
	Mcp1	AACTTTATTTAAAACTGCATCTGCC	123	NM_011333.3
		CCTACAGAAGTGCTTGAGGTG		
	Il-1β	TGCCACCTTTTGACAGTGATG	138	NM_008361.4
		TGATGTGCTGCTGCGAGATT		
	α-Sma	GTTCAGTGGTGCCTCTGTCA	101	NM_007392.3
		ACTGGGACGACATGGAAAAG		
	Timp1	TCTCTAGGAGCCCCGATCTG	66	NM_011593.2
		GCATGGACATTTATTCTCCACTGT		
	Tgf-β	CTCCCGTGGCTTCTAGTGC	133	NM_011577.2
		GCCTTAGTTTGGACAGGATCTG		
	Col1a1	GATGACGTGCAATGCAATGAA	122	NM_007742.4
		CCCTCGACTCCTACATCTTCTGA		
	Bip	CAGCCAATTATCAGCAAACTCT	205	NM_022310.3
		CAACTCCACTCTGAGGTGAAG		
	Atf4	CCTTCGACCAGTCGGGTTTG	189	NM_009716.3
		CTGTCCCGGAAAAGGCATCC		
	Atf6	GAAGACTGGGAGTCGACGTT	139	NM_001081304.1
		ACTCCCAAGGCATCAAATCCAA		
	Ire1	CAGGGTCGAGACAAACAACA	141	NM_023913.2
		GAGTCAGGGCCACGTCCT		
	Chop	CGGAACCTGAGGAGAGAGTG	121	NM_007837.4
		GTCTCCAAGGTGAAAGGCAG		
	Asgr1	CTGCCAGATGGCTGCATTTC	116	NM_009714.3
		CAGTCCAAGGCCTCACAGAG		
	Caspase-3	GAAACTCTTCATCATTCAGGCC	250	NM_001284409.1
		GCGAGTGAGAATGTGCATAAAT		
	Caspase-9	TGTGAATATCTTCAACGGGAGC	249	NM_001277932.1
		GAGTAGGACACAAGGATGTCAC		
	Bax	TTGCCCTCTTCTACTTTGCTAG	81	NM_007527.3
		CCATGATGGTTCTGATCAGCTC		
	Bcl-2	GATGACTTCTCTCGTCGCTAC	156	NM_009741.5
		GAACTCAAAGAAGGCCACAATC		
	Cyclin A2	CTTGGCTGCACCAACAGTAA	109	NM_009828.3
		CAAACTCAGTTCTCCCAAAAACA		
	Cyclin B1	GCGTGTGCCTGTGACAGTTA	135	NM_172301.3
		CCTAGCGTTTTTGCTTCCCTT		

Species	Gene	Primers	Size (bp)	Accession Number
Mouse	*Cyclin D1*	GCGTACCCTGACACCAATCTC	94	NM_007631.3
		ACTTGAAGTAAGATACGGAGGGC		
	Cyclin E1	TCCACGCATGCTGAATTATC	95	NM_007633.2
		TTGCAAGACCCAGATGAAGA		
	Cyp2e1	CGTTGCCTTGCTTGTCTGGA	105	NM_021282.3
		AAGAAAGGAATTGGGAAAGGTCC		
	β-actin	CGGTTCCGATGCCCTGAGGCTCTT	100	NM_007393.5
		CGTCACACTTCATGATGGAATTGA		
human	*ASGR1*	GACACGCAGACACAGAGACA	188	NM_001671.5
		CTGGACCTGGGATTGCTCAG		
	BIP	CCGCTGAGGCTTATTTGGGA	91	NM_005347.5
		TCTTTGGTTGCTTGGCGTTG		
	ATF4	ATGACCGAAATGAGCTTCCTG	152	NM_001675.4
		CTGGAGAACCCATGAGGTTTG		
	ATF6	TGTGGTCTTGTTATGGGTGG	124	NM_007348.4
		TGCTTCACCCAGAAGTTATCA		
	IRE1	ATTGTGTACCGGGGCATGTT	162	NM_001433.5
		CTGGAATTGCCGGTCCTTCT		
	CHOP	GCGCATGAAGGAGAAAGAAC	138	NM_001195053.1
		ACCATTCGGTCAATCAGAGC		
	FURIN	CAAGACCCTCACGTCCAGTC	78	NM_002569.4
		TGCTGGACACAGCTCTTCTG		
	GAPDH	AGAAGGCTGGGGCTCATTTG	258	NM_002046.7
		AGGGGCCATCCACAGTCTTC		

Table S3 Primary antibodies used in this study

Antibody	Dilution (WB/IF/IP)	Source	Cat. No
α-TUBULIN	1:4000/no/no	Proteintech	11224-1-AP
ASGR1	1:1000/1:100/1:50	Proteintech	11739-1-AP
α-SMA	1:1000/no/no	Proteintech	14395-1-AP
BIP	1:1000/1:100/no	Proteintech	66574-1-Ig
ATF4	1:1000/no/no	Proteintech	10835-1-AP
ATF6	1:1000/no/no	Santa Cruz	sc-166659
IRE1	1:1000/no/no	Santa Cruz	sc-390960
p-IRE1	1:1000/no/no	ABclonal	AP0878
CHOP	1:1000/1:100/no	Proteintech	15204-1-AP
CD11b	no/1:100/no	Servicebio	GB11058
LAMP1	no/1:100/no	Proteintech	67300-1-Ig
CYP2E1	1:1000/no/no	Proteintech	19937-1-AP
Ki67	no/1:100/no	Servicebio	GB151141
ATP1A1	no/1:100/no	Proteintech	14418-1-AP
FURIN	1:1000/no/no	Santa Cruz	sc-133142
Anti-Rabbit IgG	1:10000 for WB	Abbkine	A21020
Anti-Mouse IgG	1:10000 for WB	Abbkine	A21010
