# Supplementary material for: Deficiency of ASGR1 promotes liver injury by increasing GP73-mediated hepatic endoplasmic reticulum stress
Source: Nat Commun. 2024 Mar 8;15:1908. doi: 10.1038/s41467-024-46135-9 (PMC10924105; doi:10.1038/s41467-024-46135-9)
Supplement: Supplementary file 3 — Reporting Summary [file 41467_2024_46135_MOESM3_ESM.pdf]

nature portfolio

Corresponding author(s): Xiao Gang Zhang, Kai Shan Tao and Jiang Wei Wu

Last updated by author(s): Jan 19, 2024

Reporting Summary

Nature Portfolio wishes to improve the reproducibility of the work that we publish. This form provides structure for consistency and transparency in reporting. For further information on Nature Portfolio policies, see our Editorial Policies and the Editorial Policy Checklist.

Statistics

For all statistical analyses, confirm that the following items are present in the figure legend, table legend, main text, or Methods section.

n/a	Confirmed	
☐	☒	The exact sample size (n) for each experimental group/condition, given as a discrete number and unit of measurement
☐	☒	A statement on whether measurements were taken from distinct samples or whether the same sample was measured repeatedly
☐	☒	The statistical test(s) used AND whether they are one- or two-sided<br>*Only common tests should be described solely by name; describe more complex techniques in the Methods section.*
☒	☐	A description of all covariates tested
☐	☒	A description of any assumptions or corrections, such as tests of normality and adjustment for multiple comparisons
☐	☒	A full description of the statistical parameters including central tendency (e.g. means) or other basic estimates (e.g. regression coefficient) AND variation (e.g. standard deviation) or associated estimates of uncertainty (e.g. confidence intervals)
☐	☒	For null hypothesis testing, the test statistic (e.g. F, t, r) with confidence intervals, effect sizes, degrees of freedom and P value noted<br>*Give P values as exact values whenever suitable.*
☒	☐	For Bayesian analysis, information on the choice of priors and Markov chain Monte Carlo settings
☒	☐	For hierarchical and complex designs, identification of the appropriate level for tests and full reporting of outcomes
☐	☒	Estimates of effect sizes (e.g. Cohen's d, Pearson's r), indicating how they were calculated

Our web collection on statistics for biologists contains articles on many of the points above.

Software and code

Policy information about availability of computer code

Data collection	Confocal microscopy: Leica TCS SP8 Laser Scanning Confocal Microscope
Data analysis	GraphPad Prism version 8.0.2; Image Jversion 1.8.0.

For manuscripts utilizing custom algorithms or software that are central to the research but not yet described in published literature, software must be made available to editors and reviewers. We strongly encourage code deposition in a community repository (e.g. GitHub). See the Nature Portfolio guidelines for submitting code & software for further information.

Data

Policy information about availability of data

All manuscripts must include a data availability statement. This statement should provide the following information, where applicable:

- Accession codes, unique identifiers, or web links for publicly available datasets
- A description of any restrictions on data availability
- For clinical datasets or third party data, please ensure that the statement adheres to our policy

All the sequencing data have been submitted to the NCBI’s Gene Expression Omnibus data bank and the access number is GSE232677. All data needed to evaluate the conclusions in the paper are present in the paper and/or the Supplementary Materials. All data supporting the findings of this study are avaliable in the Source Data file.

Research involving human participants, their data, or biological material

Policy information about studies with human participants or human data. See also policy information about sex, gender (identity/presentation), and sexual orientation and race, ethnicity and racism.

Reporting on sex and gender	Liver tissues and serum samples were obtained from 24 females and 25 males at Xijing Hospital of the Air Force Medical University (sex of participants was determined based on their sex at birth). Sample sizes of human participants precluded sex-based subset analysis.
Reporting on race, ethnicity, or other socially relevant groupings	The samples are all from Chinese Han population.
Population characteristics	Liver tissue and serum samples were obtained from 15 healthy living donors (sex, male: n=8, female: n=7; age: 30-52 (median= 40.27)) , 6 patients with liver fibrosis (sex, male: n=3, female: n=3; age: 44-57 (median= 50.16)), 10 patients with liver cirrhosis (sex, male: n=4, female: n=6; age: 46-65 (median= 55)) and 18 patients with hepatocellular carcinoma (sex, male: n=10, female: n=8; age: 37-78 (median= 60.11); stage, stage 1: n=6, stage 2: n=6, stage 3: n=6). All liver tissue and serum samples were obtained at Xijing Hospital of the Air Force Medical University.
Recruitment	All the participants in this study were enrolled at Xijing Hospital of the Air Force Medical University. Patients were recruited according to the etiology and the severity of liver disease. The inclusion criteria for this study were: 1. pathological diagnosis of liver cirrhosis, liver fibrosis or liver cancer; 2. complete clinical data and follow-up information; 3. specimens available. Exclusion criteria included: 1. age< 18 years old; 2. concurrent with other tumors; 3. clinically diagnosed as B or C cirrhosis. Informed written consents were obtained from all participants.
Ethics oversight	The present study was approved by the Ethics Committee of Xijing Hospital of the Air Force Medical University (approval number: KY20172013-1 and KY20232280-X-1).

Note that full information on the approval of the study protocol must also be provided in the manuscript.

Field-specific reporting

Please select the one below that is the best fit for your research. If you are not sure, read the appropriate sections before making your selection.

- [x] Life sciences
- [ ] Behavioural & social sciences
- [ ] Ecological, evolutionary & environmental sciences

For a reference copy of the document with all sections, see nature.com/documents/nr-reporting-summary-flat.pdf

Life sciences study design

All studies must disclose on these points even when the disclosure is negative.

Sample size	No statistical test was used to predetermine sample size. Sample size was based on experimental feasibility, sample availability, and necessary to obtain definitive results.
Data exclusions	No data were excluded.
Replication	Experiments were repeated with the same conditions and obtained similar results. The numbers of repeats were indicated in figure legends.
Randomization	All samples were randomly allocated.
Blinding	Blinds are placed on all participants in the experiment, including researchers, technicians, data analysts, and evaluators.

Reporting for specific materials, systems and methods

We require information from authors about some types of materials, experimental systems and methods used in many studies. Here, indicate whether each material, system or method listed is relevant to your study. If you are not sure if a list item applies to your research, read the appropriate section before selecting a response.

Materials & experimental systems

n/a	Involved in the study
[ ]	[x] Antibodies
[ ]	[x] Eukaryotic cell lines
[x]	[ ] Palaeontology and archaeology
[ ]	[x] Animals and other organisms
[x]	[ ] Clinical data
[x]	[ ] Dual use research of concern
[x]	[ ] Plants

Methods

n/a	Involved in the study
[x]	[ ] ChIP-seq
[x]	[ ] Flow cytometry
[x]	[ ] MRI-based neuroimaging

Antibodies

Antibodies used

Rabbit polyclonal Anti-α-TUBULIN (Proteintech, 11224-1-AP, 1:4000 for WB);
Rabbit polyclonal Anti-ASGR1 (Proteintech, 11739-1-AP, 1:1000 for WB, 1:100 for IF, 1:50 for IP);
Rabbit monoclonal Anti-α-SMA (Proteintech, 14395-1-AP, 1:1000 for WB);
Mouse Monoclonal Anti-BIP (Proteintech, 66574-1-Ig, 1:1000 for WB, 1:100 for IF);
Rabbit polyclonal Anti-ATF4 (Proteintech, 10835-1-AP, 1:1000 for WB);
Mouse Monoclonal Anti-ATF6 (Santa Cruz, sc-166659, 1:1000 for WB);
Mouse Monoclonal Anti-IRE1 (Santa Cruz, sc-390960, 1:4000 for WB);
Rabbit monoclonal Anti-p-IRE1 (ABclonal, AP0878, 1:1000 for WB);
Rabbit polyclonal Anti-CHOP (Proteintech, 15204-1-AP, 1:1000 for WB, 1:100 for IF);
Rabbit polyclonal Anti-CD11b (Servicebio, GB11058, 1:100 for IF);
Mouse Monoclonal Anti-LAMP1 (Proteintech, 67300-1-Ig, 1:100 for IF);
Mouse Monoclonal Anti-FURIN (Santa Cruz, sc-133142, 1:1000 for WB);
Rabbit polyclonal Anti-ATP1A1 (Proteintech, 14418-1-AP, 1:100 for IF);
Rabbit polyclonal Anti-CYP2E1 (Proteintech, 19937-1-AP, 1:1000 for WB);
Mouse Monoclonal Anti-Ki67 (Servicebio, GB121141, 1:100 for IF);
Goat Anti-Rabbit IgG (Abbkine, A21020, 1:10000 for WB);
Goat Anti-Mouse IgG (Abbkine, A21010, 1:10000 for WB).

Validation

Rabbit polyclonal Anti-α-TUBULIN (Proteintech, 11224-1-AP, https://www.ptgcn.com/products/TUBA1B-Antibody-11224-1-AP.htm);
Rabbit polyclonal Anti-ASGR1 (Proteintech, 11739-1-AP, https://www.ptgcn.com/products/ASGR1-Antibody-11739-1-AP.htm);
Rabbit polyclonal Anti-α-SMA (Proteintech, 14395-1-AP, https://www.ptgcn.com/products/ACTA2-Antibody-14395-1-AP.htm);
Mouse Monoclonal Anti-BIP (Proteintech, 66574-1-Ig, https://www.ptgcn.com/products/GRP78,BIP-Antibody-66574-1-Ig.htm);
Rabbit polyclonal Anti-ATF4 (Proteintech, 10835-1-AP, https://www.ptgcn.com/products/ATF4-Antibody-10835-1-AP.htm);
Mouse Monoclonal Anti-ATF6 (Santa Cruz, sc-166659, https://www.scbt.com/p/atf-6alpha-antibody-f-7?requestFrom=search);
Mouse Monoclonal Anti-IRE1 (Santa Cruz, sc-390960, https://www.scbt.com/p/ire1alpha-antibody-b-12?requestFrom=search);
Rabbit monoclonal Anti-p-IRE1 (ABclonal, AP0878, https://abclonal.com/catalog-antibodies/PhosphoIRE1S724RabbitpAb/AP0878);
Rabbit polyclonal Anti-CHOP (Proteintech, 15204-1-AP, https://www.ptgcn.com/products/DDIT3-Antibody-15204-1-AP.htm);
Rabbit polyclonal Anti-CD11b (Servicebio, GB11058, https://www.servicebio.cn/goodsdetail?id=1339);
Mouse Monoclonal Anti-LAMP1 (Proteintech, 67300-1-Ig, https://www.ptgcn.com/products/CD107a-Antibody-67300-1-Ig.htm);
Mouse Monoclonal Anti-FURIN (Santa Cruz, sc-133142, https://www.scbt.com/zh/p/furin-antibody-b-6);
Rabbit polyclonal Anti-ATP1A1 (Proteintech, 14418-1-AP, https://www.ptgcn.com/products/ATP1A1-Antibody-14418-1-AP.htm);
Rabbit polyclonal Anti-CYP2E1 (Proteintech, 19937-1-AP, https://www.ptgcn.com/products/CYP2E1-Specific-Antibody-19937-1-AP.htm);
Mouse Monoclonal Anti-Ki67 (Servicebio, GB121141, https://www.servicebio.cn/goodsdetail?id=6801);
Goat Anti-Rabbit IgG (Abbkine, A21020, https://www.abbkine.cn/product/a21020/);
Goat Anti-Mouse IgG (Abbkine, A21010, https://www.abbkine.cn/product/a21010/).

Eukaryotic cell lines

Policy information about cell lines and Sex and Gender in Research

Cell line source(s)	HepG2 cells were purchased from American Type Culture Collection (ATCC).
Authentication	HepG2 is a cell line exhibiting epithelial-like morphology that was isolated from a hepatocellular carcinoma of a 15-year-old, White, male youth with liver cancer. The cell line was obtained from suppliers. Cell authentication is based on their morphology, growth conditions and specific gene expression.
Mycoplasma contamination	We routinely check for mycoplasma in the lab and the cells were confirmed mycoplasma free at time of analysis.
Commonly misidentified lines (See ICLAC register)	No commonly misidentified cell lines were used in this study.

Animals and other research organisms

Policy information about studies involving animals; ARRIVE guidelines recommended for reporting animal research, and Sex and Gender in Research

Laboratory animals	ASGR1-deficient mice were purchased from Cyagen Biosciences (Guangzhou, China). C57BL/6J mice were obtained from the animal center of Xi'An Jiao Tong University (Xi'an, China). 8-week-old or 6-month-old ASGR1-deficient mice and their corresponding controls were used in this study. All mice were housed in the animal facility at Northwest A&F University under standard conditions with free access to food and water. The light was on from 7am to 7pm, with the temperature kept at 21-24 ℃ and humidity at 40-70%.
Wild animals	This study did not involve wild animals.
Reporting on sex	It has been reported that female mice were resistant to the hepatotoxic effects of APAP than male mice. Considering this, male mice were used in this study.
Field-collected samples	This study did not involve samples collected from the field.
Ethics oversight	All mouse experiments were approved by the Animal Ethical and Welfare Committee of Northwest A&F University (Yangling, China) (NWAFU-314023743).

Note that full information on the approval of the study protocol must also be provided in the manuscript.
